# White paper on the current state of imaging biomarkers in paediatric oncology

**DOI:** 10.1007/s00247-025-06372-6

**Published:** 2025-09-02

**Authors:** Rutger A.J. Nievelstein, Lise Borgwardt, Emilio J. Inarejos Clemente, Thekla Von Kalle, Martin Kyncl, Maarten H. Lequin, Annemieke S. Littooij, Erika Pace, Pier L. Di Paolo, Rick R. Van Rijn, Julian M.M. Rogasch, Jurgen Schäfer, Irmina Sefic Pasic, Anita Spezzacatene, Nelleke Tolboom, Simon M. Wan, Pietro Zucchetta

**Affiliations:** 1 Department of Radiology and Nuclear Medicine, Division Imaging and Oncology, University Medical Center Utrecht, Wilhelmina Children’s Hospital, P.O. Box 85500, 3508 GA Utrecht, Netherlands; 2 Department of Radiology and Nuclear Medicine, Princess Máxima Center for Pediatric Oncology, Utrecht, Netherlands; 3 Department of Clinical Physiology and Nuclear Medicine, Copenhagen University Hospital - Rigshospitalet, Copenhagen, Denmark; 4 Department of Diagnostic Imaging, Nuclear Medicine and Interventional Radiology, Sant Joan de Déu Barcelona Children’s Hospital, Barcelona, Spain; 5 Radiological Institute, Olgahospital, Klinikum Stuttgart, Stuttgart, Germany; 6 Department of Radiology and Center for Pediatric Neuro-Oncology, Second Faculty of Medicine, Charles University Prague and University Hospital in Motol, Prague, Czech Republic; 7 Edward B. Singleton Department of Radiology, Texas Children’s Hospital and Baylor College of Medicine, Houston, United States; 8 Department of Radiology, Royal Marsden NHS Foundation Trust and Institute of Cancer Research, London, United Kingdom; 9 Department of Radiology, Bambino Gesù Children’s Hospital, Rome, Italy; 10 Department of Radiology and Nuclear Medicine, Amsterdam University Medical Center, Amsterdam, Netherlands; 11 Department of Nuclear Medicine, Charité - University Medicine Berlin, Berlin, Germany; 12 Division of Pediatric Radiology, Department of Radiology, Universitätsklinikum Tübingen, Tübingen, Germany; 13 Department of Radiology, University Medical Center Maribor, Maribor, Slovenia; 14 Radiology Department, IRCCS Materno Infantile Burlo Garofolo, Trieste, Italy; 15 Department of Radiology and Nuclear Medicine, Division Imaging and Oncology, University Medical Center Utrecht, Utrecht, Netherlands; 16 Institute of Nuclear Medicine, University College London Hospitals NHS Foundation Trust, London, United Kingdom; 17 Nuclear Medicine Department, University Hospital of Padua, Padua, Italy

**Keywords:** Biomarkers, Child, Diagnostic imaging, Nuclear medicine, Radiology, Oncology, Radionuclide imaging

## Abstract

In paediatric oncology, imaging biomarkers play an increasing role in diagnostic imaging and research. They can be used for prediction, detection, staging, and grading of diseases, as well as for assessment of response to treatment. Imaging biomarkers are complementary to tissue-based biomarkers, enabling a more personalised approach in oncology care. In this white paper by the European Society of Paediatric Radiology (ESPR) Oncology Taskforce and European Association of Nuclear Medicine (EANM) Paediatrics Committee, an overview is given of the current knowledge on the use of imaging biomarkers in general and per tumour group.

**Graphical Abstract:**

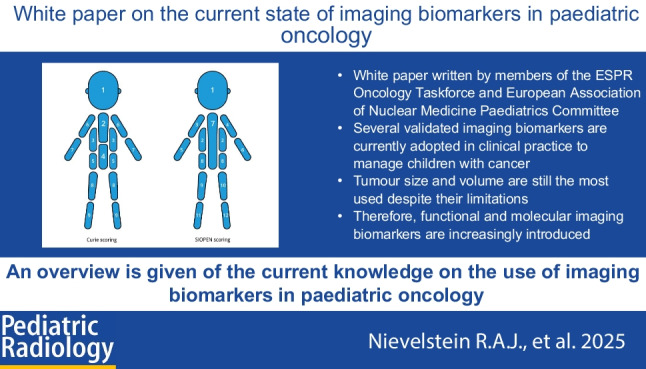

## Introduction

Imaging biomarkers play an increasing role in cancer, both from a clinical point of view and in research. They are indicators of normal/pathological processes or a (pharmacological) response to a therapeutic intervention which can be objectively measured [1]. Imaging biomarkers can be used for prediction, detection, staging, grading of diseases, and assessment of treatment response. Compared to tissue-based characteristics, they have the advantage of being non-invasive without any risk of sampling errors or limits in frequency of evaluation during treatment; therefore, they are complementary to the former, enabling a personalised approach in oncology care [2].

Notwithstanding that several imaging biomarkers are already extensively adopted in daily clinical practice or do show great potential, most are still lacking a consistent validation and qualification process necessary to become a medical research or clinical decision-making tool. This is especially true in paediatric oncology. Recently, representatives from Cancer Research United Kingdom (CRUK) and the European Organisation for Research and Treatment of Cancer (EORTC), together with other assembled experts in cancer imaging sciences, oncology, biomarker development, biostatistics, and health economics, have formulated an imaging biomarker roadmap for cancer studies [3]. In this consensus statement, specific considerations for validation and qualification of these biological characteristics are described alongside recommendations to accelerate the successful clinical translation of effective indicators and discontinuation of the fruitless ones.

With this white paper, the Oncology Taskforce of the European Society of Paediatric Radiology (ESPR) and Paediatrics Committee of the European Association of Nuclear Medicine (EANM) aim to further intensify the collaboration on research projects, and validation and implementation of imaging biomarkers in paediatric oncology. In this article, an overview of the current knowledge on the use of these indicators per tumour group is provided.

## Types of imaging biomarkers

Nowadays, several anatomical, functional, and molecular imaging biomarkers are available. To date, anatomical imaging biomarkers, in particular tumour size or volume, are the most adopted and often only imaging surrogate endpoints accepted both in oncological trials and clinical practice, as defined in the “Response Evaluation Criteria in Solid Tumours” (RECIST), version 1.1; “Response Assessment in Neuro-oncology Criteria” (RANO); and “Lugano classification” for lymphoma [4–6]. Although mainly applied in adult and increasingly used in paediatric oncology, they are most likely not completely suitable for children. Therefore, modified tools for children, such as Pediatric Response Evaluation Criteria in Solid Tumours (Ped-RECIST) and Response Assessment in Pediatric Neuro-Oncology (RAPNO), have recently been proposed [7, 8]. Of note, these size-based criteria are already frequently used in clinical trials and practice for (re)staging and treatment stratification, even though studies on variability depending on tumour shape, location, imaging device, and observer are lacking [9].

Another major disadvantage of anatomical imaging biomarkers is that they do not reflect intratumoural architectural changes due to the treatment, such as necrosis or cystic degeneration. Functional imaging biomarkers probe important hallmarks of disease, including apoptosis, cell proliferation, tumour invasiveness, angiogenesis, glycolysis, hypoxia, inflammation, and fibrosis. They can be obtained and evaluated by magnetic resonance imaging (MRI) through contrast-enhancement (CE), perfusion-weighting (PWI), diffusion-weighting (DWI), elastography, and spectroscopy (MRS), as well as by positron emission tomography (PET) and single-photon emission computed tomography (SPECT).

Finally, in addition to the clinically used molecular imaging biomarkers 2-deoxy-2[^18^F]fluoro-d-glucose ([^18^F]FDG) and ^123^iodine-labeled metaiodobenzylguanidine ([^123^I]mIBG), newer molecular imaging biomarkers, encompassing copious small target-specific radiotracers, are under investigation in preclinical research settings, further aiding visualisation, characterisation, and measurement of biological activities in patients.

## Tumour groups

**Fig. 1:**
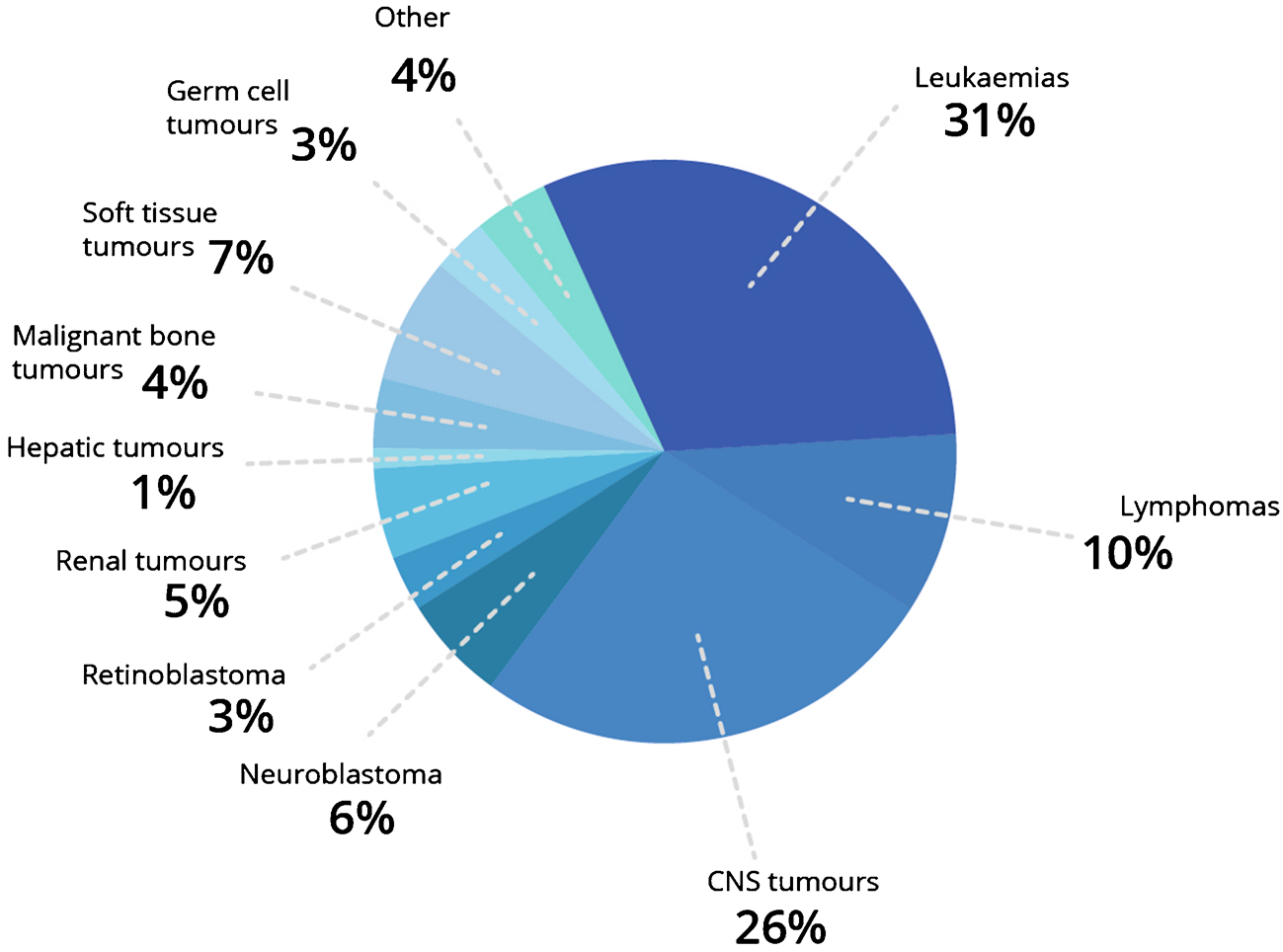
Main types of cancers in children [based on 10]

In this part of the paper, we will focus on specific groups of paediatric tumours (Fig. 1), as there are differences in the use of and evidential foundation for imaging biomarkers in the different paediatric tumour groups.

### Neoplasms of the central nervous system

Up to 26% of paediatric malignancies arise from the central nervous system [10]. Advances in the molecular-genetic characterisation led to their modern reclassification integrating imaging, histology, immunohistochemistry, and molecular biology [11]. Although the diagnosis, prognosis, and treatment are now highly reliant on the lesion’s histopathologic features and genetic profile, MRI plays a crucial role in narrowing the differential diagnosis, assessing treatment response, and sometimes predicting the tumour subtype and prognosis [12]. Imaging can predict molecular subtypes of medulloblastoma based on the anatomical site, saturation after gadolinium-based contrast agents, and the presence of metastases. For example, lateral cerebellar hemispheric medulloblastomas usually belong to the sonic hedgehog (SHH) subgroup, whereas the location of posterior cranial fossa ependymomas and low-grade gliomas aids differentiating the (molecular) subgroups [13–15].

DWI is part of a standard MRI protocol, especially in oncological settings, with restricted diffusion being associated with malignancy and apparent diffusion coefficient (ADC) being a valid imaging biomarker. Its coefficients can be adopted to distinguish medulloblastoma from other neoplastic entities in the posterior fossa [16]. In addition, BRAF V600E mutant gangliogliomas and pilocytic astrocytomas seem to have lower relative ADCmean and ADCmin values than wild type, suggesting a radiogenomic correlation and molecular profile [17].

A relatively new imaging biomarker, the T2-FLAIR-mismatch sign, appears to be an insensitive but highly specific indicator of isocitrate dehydrogenase (IDH) mutation but not 1p/19q codeletion in diffuse low-grade glioma in adults [18]. In the paediatric population, this mismatch is more recognised in MYB and MYBL1 altered diffuse astrocytoma, which have a better prognosis than the former. Moreover, magnetic resonance spectroscopy (MRS) may help to differentiate among several molecular subgroups of medulloblastoma. In a study by Blüml et al., it was shown that group 3/4 medulloblastomas demonstrated a readily detectable taurine peak, high levels of creatine, and lower levels of lipids [19]. On the contrary, sonic hedgehog (SHH) medulloblastomas show prominent choline and lipid peaks, low levels of creatine, and little or no evidence of taurine [19].

Amino acid PET, such as O-(2-[^18^F]fluoroethyl)-l-tyrosine ([^18^F]FET), has been shown to help discriminate tumours from benign lesions in a large cohort of children and to support difficult clinical decision-making (including change of treatment strategy and biopsy guidance) [20, 21]. Joint PET recommendations by the European Association of Nuclear Medicine (EANM), European Society of Paediatric Oncology (SIOPE), Response Assessment in Pediatric Neuro-Oncology (RAPNO) working group, and Society of Nuclear Medicine and Molecular Imaging (SNMMI) on paediatric gliomas have recently been published [22].

The Response Assessment in Pediatric Neuro-Oncology (RAPNO) working group has developed standardised criteria for estimating the effect of treatments on low- and high-grade gliomas, medulloblastoma, diffuse midline glioma, ependymoma, and craniopharyngioma based on neoplastic dimensions on MRI and clinical assessment [8, 23–28]. These have been instrumental in addressing challenging cases and could improve the consistency and accuracy of disease evaluation in patients enrolled onto clinical trials.

### Lymphoma

The lymphomas are the third most frequent paediatric cancer, with Hodgkin lymphoma (HL) predominantly affecting teenagers and non-Hodgkin variants (NHL) affecting more individuals younger than 15 years [10]. The Lugano classification alongside Deauville scores (as defined by the European Network-Paediatric Hodgkin Lymphoma Study Group (EuroNet-PHL)) and the International Pediatric Non-Hodgkin Lymphoma Staging System (IPNHLSS) are adopted for staging them, respectively [6, 29, 30].

In Hodgkin lymphoma, [^18^F]FDG PET (combined with computed tomography (CT) or MRI) is the modality of choice for staging and treatment response assessment, providing both structural and functional metabolic information [31]. Preferably, only low-dose CT for attenuation correction (instead of diagnostic contrast-enhanced CT) should be acquired to limit the radiation exposure; also, radioactivity dosages can be lessened thanks to the new and more sensitive (total body) PET scanners. This is very important because children are more sensitive to ionising radiation than adults with a potential but very low risk of adverse effects, in particular the induction of cancer later during life. There is an increasing interest in and validation of the use of whole-body (WB)-MRI as a radiation-free alternative, at least for staging purposes [32]. In non-Hodgkin lymphoma, imaging recommendations are still more heterogeneous depending on the subtype and often still include chest X-ray, ultrasound of the abdomen, and cranial/spinal MRI if indicated. Only in B-cell non-Hodgkin lymphoma, [^18^F]FDG PET/CT and/or WB-MRI are increasingly used [33, 34].

In Hodgkin lymphoma, tumour involvement is diagnosed with [^18^F]FDG PET/CT with very high sensitivity and specificity, and early treatment response assessment reliably predicts prognosis [31]. In fact, chemotherapy adaptation has recently been introduced, as part of the large multicentric European Network-Paediatric Hodgkin Lymphoma Study Group (EuroNet-PHL) C2 trial [29]. For response assessment, the visual Deauville scale is employed; this imaging biomarker defines the metabolic areas within the lesions compared to physiological uptake in liver and blood pool (Tables 1 and 2) [31, 35]. Furthermore, given the high accuracy of PET/CT in detecting bone marrow involvement in Hodgkin lymphoma, biopsy is currently avoided in most centres [36]. Contrary to recommendations in paediatric Hodgkin lymphoma as well as adult non-Hodgkin lymphoma, interim [^18^F]FDG PET/CT has not been validated yet as a predictive endpoint in most subtypes of paediatric non-Hodgkin lymphoma, and its use in this setting still remains mostly investigational [33, 34, 37].

**Table 1:** Deauville score criteria for interim [^18^F]FDG PET/CT (adapted from [35])

Scale	Uptake
1	None
2	≤ Mediastinum
3	> Mediastinum but ≤ liver
4	Moderately > liver
5	Significantly > liver or new disease foci

**Table 2:** Response criteria in Hodgkin lymphoma (adapted from [6]

Response	PET-CT	CT, MRI
Complete	Deauville score 1, 2, or 3 with or without a residual mass No [^18^F]FDG-avid bone marrow disease No new lesion	Target nodes width/nodal masses width/LDi ≤ 1.5 cm No extra-nodal disease sites No non-measured lesion Normal bone marrow morphology No new lesion
Partial	Deauville score 4 or 5 with reduced uptake compared with baseline and residual mass(es) of any size At interim: responding disease At end of treatment: residual disease Bone marrow: residual uptake higher than uptake in normal marrow but reduced compared with baseline	≥ 50% decrease in SPPD of up to 6 target measurable nodes and extra-nodal lesions Spleen > 50% shrinkage in bipolar length beyond normal Absent/normal/regressed non-measured lesions No new lesions Bone marrow: NA
No response, stable	Deauville score 4 or 5 with no significant change in [^18^F]FDG-uptake from baseline at interim or end of treatment Bone marrow: no change from baseline No new lesions	< 50% decrease from baseline in SPPD of up to 6 target measurable nodes and extra-nodal lesions No criteria for progressive disease are met Non-measured lesions: no increase consistent with progression Bone marrow: NA No new lesions
Progression	Deauville score 4 or 5 with an increase in intensity of uptake from baseline and/or New [^18^F]FDG-avid foci consistent with disease at interim or end-of-treatment assessment	PPD progression of target measurable nodes and extra-nodal sites Individual nodes/lesions must be abnormal with: LDi ≥ 1.5 cm and Increase by ≥ 50% from SPPD nadir and Increase in LDi or SDi from nadir 0.5 cm for lesions ≤ 2 cm 1 cm for lesions > 2 cm In the setting of splenomegaly, the splenic length must increase by > 50% of the extent of its prior increase beyond baseline. If no prior splenomegaly, increase by at least 2 cm from baseline New/progression of pre-existing non-measured lesions Regrowth of previously resolved lesions A new node > 1.5 cm in any axis A new extra-nodal site > 1 cm in any axis Assessable disease of any size unequivocally attributable to lymphoma New/recurrent bone marrow involvement

*cm* centimetre, *LDi* longest transverse diameter of a lesion,* [*^*18*^*F]FDG* 2-deoxy-2 [^18^F]fluoro-d-glucose, *NA* not applicable, *PPD* cross product of the LDi and perpendicular diameter, *SDi* shortest axis perpendicular to the LDi, *SPPD* sum of the product of the perpendicular diameters for multiple lesions

Staging and chemotherapy response evaluation with WB-MRI are based on size criteria (lymph nodes, spleen, extra-nodal lesions) and imaging features (Tables 2 and 3). Several studies including paediatric patients with Hodgkin lymphoma have shown that DWI has an excellent agreement with an [^18^F]FDG PET/CT for staging, but the former is less accurate for early response to chemotherapy and restaging after it if ADC values are not included, given the fact that lymph nodes and spleen are intrinsically high-cellular [38–43]. In this scope, the use of integrated WB-PET/MRI in young patients seems interesting, combining the higher anatomical detail with the functional information of both modalities whilst decreasing the radiation dose [43–45]. However, due to the recent technical developments in (total body) PET/CT including artificial intelligence (AI)–driven reconstructions of (attenuation correction) CT, radiation exposure will not be a real issue in the near future [46, 47].

**Table 3:** Criteria for nodal and extra-nodal involvement at WB-MRI in lymphoma (adapted from [38])

Site	Definition
Nodal: - Lymph nodes - Spleen	Longest diameter > 1.5 cm or shortest diameter > 1 cm Discrete nodules, low signal on T2-weighted MRI and DWI, or enlargement (coronal bipolar length > 13 cm)
Extra-nodal: - Liver - Lung - Bone marrow	Nodules, moderate hyperintense on T2-weighted MRI, separate from adjacent lymphatic mass Discrete lesion > 1 cm Hypointense T1-weighted and hyperintense T2-weighted signal intensity combined with restricted diffusion
E-lesion	Disease infiltration into extra-lymphatic structure or organ that is adjacent to a lymph node mass

*cm*, centimetre, *E-lesion* a contiguous infiltration of a lymph node mass into extra-lymphatic structures or organs, *MRI* magnetic resonance imaging, *DWI* diffusion-weighted imaging, *WB-MRI* whole-body MRI

### Neuroblastoma

This embryonal tumour originates from the sympathetic nervous system and is characterised by a highly variable clinical presentation ranging from rare spontaneous regression in infancy to unresponsive widespread disease [48, 49]. Cross-sectional imaging is necessary for accurate staging and assessing response to treatment with [^123^I]mIBG scintigraphy with SPECT/CT as the standard and most used technique to look for metastases; [^18^F]FDG PET/CT may assist in [^123^I]mIBG non-avid neuroblastomas (10%), but treatment-related reactive bone marrow sometimes hampers evaluation of this tissue during the follow-up [49–51]. More specific tracers are currently being evaluated, such as the PET-ligand variant of [^124^I]mIBG, [^18^F]mFBG, which should result in more accurate disease estimation [52–54].

Staging relies on the International Neuroblastoma Risk Group Staging System (INRGSS), developed in 2009, which combines clinical elements (including patient’s age and metastatic disease) and image-defined risk factors (IDRFs) to categorize patients into a stage L1 or L2 (localised) and M or MS (metastatic) [50]. The International Neuroblastoma Risk Group Staging System (INRGSS) guides treatment and predicts outcome, including 5-year event-free survival (very low risk if > 85%, low risk 75–85%, intermediate risk 50–75%, and high risk < 50%) [49, 50, 55].

With the introduction of the International Neuroblastoma Risk Group Staging System (INRGSS), a pre-operative evaluation was implemented. To enable standardised reporting, image-defined risk factors (IDRFs) were adopted (Table 4) [50] to describe the relationship of the primary tumour with vital structures [50]. A systematic review and meta-analysis conducted in 2020 concluded that patients with image-defined risk factors (IDRFs)-positive neuroblastoma have a significantly higher risk of incomplete surgical resection, post-operative complications, 5-year mortality, and relapse [56]. The revised International Neuroblastoma Response Criteria integrates the treatment response of all sites of disease based on a multimodal approach (in accordance with Response Evaluation Criteria in Solid Tumours (RECIST 1.1)) and [^123^I]mIBG scintigraphy or FDG PET/CT [50, 57, 58]. In summary, for the primary tumour and metastatic soft tissue (lymph node and non-lymph node) disease, treatment response is based on both RECIST criteria and [^123^I]mIBG (or FDG PET/CT in non-avid mIBG tumours) uptake. For bone (marrow) metastases, it is recommended to use the radionuclide scans for response assessment (including semiquantification as described below). In addition, bone marrow involvement is also quantitatively evaluated using bone marrow aspirates/biopsies of the iliac wings.

**Table 4:** Image-defined risk factors (IDRFs) in neuroblastic tumours (adapted from [50])

Ipsilateral tumour extension within two body compartments Neck-chest, chest-abdomen, lower trunk
Neck Tumour encasing carotid and/or vertebral artery and/or internal jugular vein Tumour extending to base of skull Tumour compressing the trachea
Cervico-thoracic junction Tumour encasing brachial plexus roots Tumour encasing subclavian vessels and/or vertebral and/or carotid artery Tumour compressing the trachea
Thorax Tumour encasing the aorta and/or major branches Tumour compressing the trachea and/or principal bronchi Lower mediastinal tumour, infiltrating the costo-vertebral junction between T9 and T12
Thoraco-abdominal Tumour encasing the aorta and/or vena cava
Abdomen/pelvis Tumour infiltrating the porta hepatis and/or the hepatoduodenal ligament Tumour encasing branches of the superior mesenteric artery at the mesenteric root Tumour encasing the origin of the coeliac axis, and/or of the superior mesenteric artery Tumour invading one or both renal pedicles Tumour encasing the aorta and/or vena cava Tumour encasing the iliac vessels Pelvic tumour crossing the sciatic notch
Intraspinal tumour extension whatever the location provided that: More than one-third of the spinal canal in the axial plane is invaded and/or the perimedullary leptomeningeal spaces are not visible and/or the spinal cord signal is abnormal
Infiltration of adjacent organs/structures Pericardium, diaphragm, kidney, liver, duodeno-pancreatic block, and mesentery
Conditions to be recorded, but not considered IDRFs Multifocal primary tumours Pleural effusion, with or without malignant cells Ascites, with or without malignant cells

**Fig. 2:**
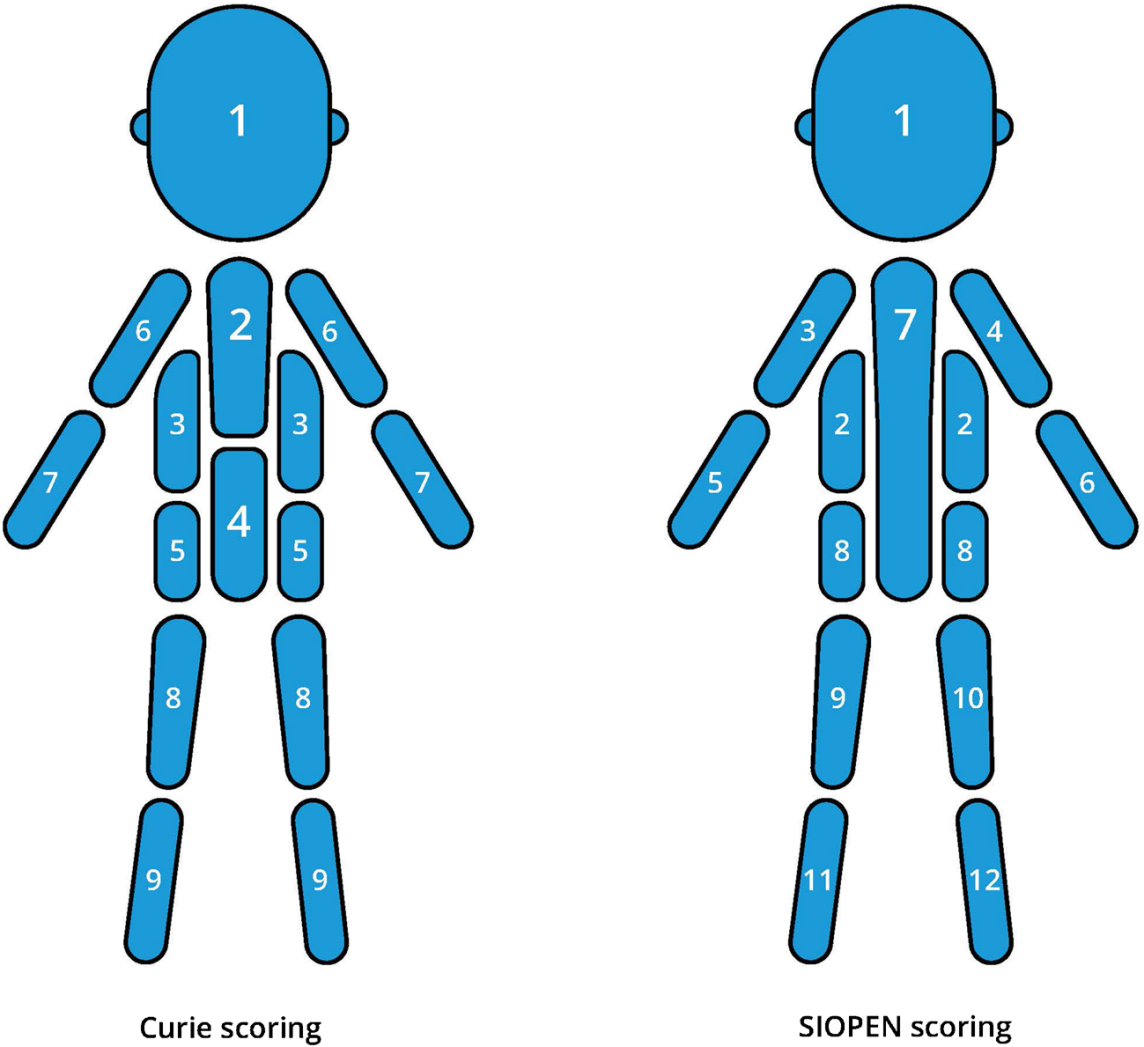
Body segments as defined for the Curie and European Society of Paediatric Oncology Neuroblastoma (*SIOPEN*) semiquantitative^123^iodine-labeled metaiodobenzylguanidine (*[*^*123*^*I]mIBG*) scoring systems for skeletal involvement in neuroblastoma. The Curie scoring system also includes a tenth segment designated for soft tissue disease, whereas the European Society of Paediatric Oncology Neuroblastoma (*SIOPEN*) scoring system only semiquantifies skeletal involvement [based on 59–61]

On planar whole-body [^123^I]mIBG scintigraphy, the validated Curie score and the European Society of Paediatric OncologyNeuroblastoma (SIOPEN) score are nowadays used to semiquantify the extent of skeletal disease (as well as soft tissue disease for the Curie score) (Table 5, Fig. 2); both are equally reliable and predictive [59–61]. Bone marrow involvement is associated with prognosis and survival in high-risk neuroblastoma patients [62]. To date, both the Curie score and the European Society of Paediatric Oncology Neuroblastoma (SIOPEN) score have been validated as prognostic biomarkers exclusively using planar [^123^I]mIBG scintigraphy images. The incorporation of SPECT or SPECT/CT imaging can significantly enhance lesion detection in [^123^I]mIBG studies, particularly for lesions located near areas of intense [^123^I]mIBG uptake which would have been overlooked on planar imaging [60]. This would systematically result in higher scores compared to planar imaging alone; however, there is currently no evidence demonstrating superior prognostic value of [^123^I]mIBG SPECT or SPECT/CT.

**Table 5:** The European Society of Paediatric Oncology Neuroblastoma (*SIOPEN*) score for skeletal involvement on planar ^123^iodine-labeled metaiodobenzylguanidine (*[*^*123*^*I]mIBG*) scintigraphy. The [^123^I]mIBG uptake over 12 body segments (Fig. 2) is scored from 0–6 points per segment [59]

Score	Definition
0	No involvement
1	One distinct spot
2	Two distinct spots
3	Three distinct spots
4	> 3 distinct lesions or diffuse involvement < 50% of the body segment
5	Diffuse involvement of 50–95% of the body segment
6	Diffuse involvement of the body segment

There are a few single-centre studies which provide evidence of ADC values as potential imaging biomarker [63, 64] to reliably distinguish among (ganglio)neuroblastoma and ganglioneuroma. However, these preliminary findings lack validation in large prospective cohort studies with standardised protocols, measurement methods, and interobserver analyses.

### Genitourinary tumours

#### Renal tumours

Wilms’ tumour or nephroblastoma is the most frequent paediatric renal tumour [65]; it can develop on the background of precursor lesions, nephrogenic rests that are more often seen in syndromes (such as Beckwith-Wiedemann, Denys-Drash, and WAGR (Wilms’ tumour, aniridia, genitourinary anomalies, retardation) syndrome) [66]. Wilms’ tumour affects one or both kidneys, has a peak age of 3 years and a half, and an excellent prognosis with overall survival rates reaching 90% [67]. They consist of variable amounts of blastemal, epithelial, and stromal elements (triphasic) ranging from poor to well-differentiated.

The International Society of Pediatric Oncology – Renal Tumour Study Group (SIOP-RTSG) does not advocate invasive procedures at diagnosis to determine the histology of a renal neoplasm in young children before starting neoadjuvant chemotherapy if the patient’s demographic data are typical and imaging findings suggestive of a non-Wilms’ tumour are absent [68, 69].

Tumour volume is an established imaging biomarker within the International Society of Pediatric Oncology – Renal Tumour Study Group (SIOP-RTSG) protocol to decide if biopsy is necessary at diagnosis in patients between 7 and 10 years old. In fact, if the renal lesion is smaller than 200 ml in a child above the age of 7 years, a biopsy is indicated; this recommendation is based on the general smaller size of renal cell carcinoma than Wilms’ tumour at presentation [70]. Furthermore, during neoadjuvant chemotherapy, an increase in size of the renal mass could necessitate histopathological confirmation either through biopsy or primary nephrectomy. In the current International Society of Pediatric Oncology – Renal Tumour Study Group (SIOP-RTSG) guidelines, the neoplastic volume has been added as a risk factor for a subgroup of Wilms’ tumour following the analyses of children enrolled into the International Society of Pediatric Oncology (SIOP) 2001 protocol [71, 72].

Several retrospective studies found an association between ADC and histopathological features along with response to pre-operative therapy [73, 74]. Axial DWI with multiple B values is advised standard of care within the current International Society of Pediatric Oncology – Renal Tumour Study Group (SIOP-RTSG) UMBRELLA protocol [75]. Moreover, intravoxel incoherent motion (IVIM) MRI allows for measurements of a wider range of diffusion metrics including perfusion; preliminary reports describe that this technique provides the best fit for Wilms’ tumour [76].

#### Ovarian tumours

Ovarian neoplasms in children are divided into germ cell, sex cord/stromal, and surface epithelial categories based on their cellular components, with mature teratoma belonging to the first group and representing the majority (70%) [77]. Germ cell neoplasms tend to be more accurately staged by the system developed by the Children’s Oncology Group (COG) [78]. In contrast, lesions with sex cord/stromal or surface epithelial origin are predominantly classified according to the International Federation of Gynaecology and Obstetrics (FIGO) method [79].

Ultrasound is often the first modality performed in the paediatric population, especially for gynaecological indications, but MRI is the gold standard for further diagnosis and staging [80].

Imaging biomarkers include measurement of the longest dimension of the solid component with the current cut-off value being 8 cm for benign tumours; higher chances for ovarian parenchymal preservation were associated with a tumour diameter lower than 10 cm [81, 82].

As in other neoplasms, DWI can be used to distinguish benign from malignant lesions, with restricted diffusion being associated with high cellularity and thereby malignancy [81, 83]. Perfusion obtained from dynamic contrast-enhanced (DCE)-MRI can be used as an imaging biomarker for the characterisation of ovarian neoplasms, with high sensitivity of 98% being demonstrated when this technique is combined with the cellularity on DWI and ADC map; malignancy is highly likely below 1 × 10–3 mm^2^/s [84, 85].

#### Testicular tumours

The paediatric testicular neoplasms are divided into germ cell and sex cord-stromal lesions. A malignant behaviour is demonstrated when yolk sac elements, Sertoli cells, or undifferentiated stromal components are present [86]. The incidence of these neoplasms raises during puberty and in the second decade of life [87]. Moreover, cryptorchism and testicular microlithiasis represent cancer risk factors [88, 89]. Testicular neoplasms are staged according to the Children’s Oncology Group (COG) system, whereas, for adolescents with metastatic germ cell tumours, the International Germ Cell Cancer Collaborative Group (IGCCCG) staging should be applied [90].

Ultrasound is the preferred modality to examine the scrotal region, and it often enables the characterisation of a mass; chest CT and lower trunk CT or MRI examinations are mandatory for staging malignant processes [91].

The assessment of the nodal involvement (width cut-off of 8 mm) is of utmost importance [92].

On DCE-MRI (or CT), malignant lesions typically show washing out [93]. Furthermore, ADC maps calculated by region of interest drawn, including the whole testicle tumour, have been reported to be the most reproducible method to differentiate benign from malignant lesions [94, 95].

Ultrasound elastography is another helpful imaging biomarker to discriminate benign from malignant (hard pattern) testicular lesions [96, 97]. Furthermore, a study to explore the role of [^18^F]FDG PET/CT for staging of seminomas and non-seminomas in adolescents and adults demonstrated higher specificity for the latter cancers and elevated sensitivity for the former [88].

### Liver tumours

Rarely, a neoplasm originates within the liver in children, but when present, two-thirds of them are malignant, with hepatoblastoma being the protagonist and the rest comprising benign lesions of epithelial or mesenchymal origin [98, 99]. Hepatoblastoma predominantly affects subjects younger than 3 years and is staged according to the PRE-Treatment EXTent of tumour (PRETEXT) system [100, 101].

A consensus paper published by the Liver Imaging Reporting and Data System (LI-RADS) working group describes the recommendations for paediatric malignant lesions, suggesting obtaining pre-operative MRI preferably with hepatobiliary contrast agents [102]. This preference is due to the increased detection and improved characterisation of liver lesions compared to traditional extracellular contrast agents.

In non-metastatic hepatoblastoma, tumour shrinkage can be adopted as an imaging biomarker after neoadjuvant chemotherapy; if this is at least 25%, the 5-year overall survival rate is 91% [103]. Furthermore, the 3-year recurrence-free survival was poorer if the tumour size reduction was less than 50% [104].

Regarding enhancement patterns, the absence of accumulation of hepatobiliary MR contrast agent (e.g. Gd-EOB-DTPA) has been described as a potential imaging biomarker for detection of hepatoblastoma and hepatocellular carcinoma [105, 106].

Elastography both on ultrasound and MRI can also be employed for the differentiation between benign and malignant liver tumours. However, it is essential to have established reference levels for normal liver parenchyma [107]. Özmen et al. in their research show higher stiffness for malignant (58—66 kPa) versus benign lesions (22–24 kPa) [108].

### Soft tissue tumours 

These heterogeneous mostly aggressive entities comprise rhabdomyosarcoma (half of the group) and non-rhabdomyosarcoma soft tissue sarcoma [109, 110]. More than one-third of rhabdomyosarcomas originates from the urinary bladder, prostate, paratesticular tissues, vagina, uterus, and cervix [111]. There are three histological types: embryonal (intermediate prognosis; with botryoid as a possible variant); alveolar (very aggressive disease, especially if tumours are PAX3-FOXO1 fusion-positive); and pleomorphic (very rare entity mainly affecting adults).

At diagnosis, high-resolution MRI of the primary tumour, chest CT, and whole-body [^18^F]FDG PET/CT (or MRI) are mandatory for staging [112, 113]. The European paediatric Soft-tissue Sarcoma Study Group (EpSSG) guidelines recommend [^18^F]FDG PET/CT at baseline and during chemotherapy in case of persistent avid lymphadenopathy or metastases until they become negative [112].

Besides the tumour location, invasion of adjacent organs, and presence of metastases, the primary lesion size is an important imaging biomarker. It has been demonstrated that masses larger than 5 cm at baseline have poorer outcomes [114]. Most soft-tissue sarcomas shrink under chemotherapy. However, unless there is progression under therapy, reduction of tumour volume is not predictive for 5-year overall survival [115]. Parameters such as ADC, perfusion (DCE-MRI), and MRS are promising imaging biomarkers for characterisation and therapy monitoring of the primary tumours.

### Bone tumours

Osteosarcoma and Ewing sarcoma are the most common paediatric bone sarcomas and have the highest incidence in the second decade of life [116].

Although radiography is the first-line examination performed to evaluate bone tumours, MRI is the modality of choice by acquiring T1- and T2-weighted sequences to assess the extent of the primary tumour and its relation to adjacent joints, nerves, and vessels [117]. Three-dimensional measurements are recommended by the European Clinical Practice Guidelines or one-dimensional measurements based on the Response Evaluation Criteria in Solid Tumours (RECIST 1.1) [116, 118]. Tumour volume at baseline is a prognostic factor for both entities. For Ewing sarcomas, the cut-off is defined at 200 ml [116]. General staging includes chest CT and bone scintigraphy [116]. However, WB-MRI and [^18^F]FDG PET/CT are increasingly used as a replacement of the latter, likely replacing the need for invasive bone marrow biopsies [116, 119, 120].

Evaluation of tumour response to neoadjuvant chemotherapy, however, still mainly relies on histological criteria which are only obtained after complete resection [116]. Monitoring size under therapy by MRI is well-applicable to the soft tissue components of Ewing sarcomas. Whereas osteosarcomas – because of their osseous matrix – typically do not shrink under chemotherapy. The latter also applies to most intraosseous parts of all bone tumours and their metastases [117].

T1-weighted sequences with fat saturation after gadolinium injection can help differentiate necrosis from viable tumour. The value of static contrast-enhanced imaging is, however, limited by hyperaemic granulation tissue, diffusion of gadolinium into the necrosis, and the influence of timing (the interval between injection and k-space acquisition). DCE-MRI allows a more detailed analysis of the internal structure and viability of lesions but has not yet been established as a diagnostic routine test [117, 121].

The changes of ADC values have been shown to reflect neoplastic response a few hours after chemotherapy and, therefore, much earlier than the standard post-operative histological assessment as confirmed by several smaller studies, but correlation to patients’ event-free and overall survivals has still to be proven [122, 123]. This approach has also been shown to depend on low ADC values of the neoplasm before therapy and is, hence, not applicable to chondroid and telangiectatic osteosarcomas [124].

[^18^F]FDG PET/CT seems to be a sensitive and reliable diagnostic tool in staging and assessment of metabolic response to treatment in patients with osteosarcoma. However, false positive findings due to inflammation at the sites of tumoural lesions should be considered [125]. Combining both DWI-MRI and [^18^F]FDG PET/CT increased the diagnostic accuracy of predicting good histologic response to 85% in a study by Byun et al. [126]. Therefore, integrated PET/MRI can play a crucial role in the skeletal tumour group.

## Conclusions

Several validated imaging biomarkers are currently adopted in clinical practice to best manage paediatric patients with cancer, as demonstrated in consolidated international recommendations and guidelines. Among these, tumour size and volume are still the most used despite their recognised limitations. Therefore, alternatives, such as functional and molecular indicators, which better reflect the neoplastic architecture, metabolic behaviour, and treatment effects, are being increasingly introduced, although adequate validation and qualification processes are needed. Given the rarity of the diseases, this can only be accomplished by multidisciplinary and international collaboration, beginning with cross-border standardisation of the acquisition parameters of the examinations and quantitative post-processing methods, implying the ability to (easily) share and analyse the data.

## Data Availability

No datasets were generated or analysed during the current study.
